# Orthopaedic Resident Practice Management and Health Policy Education: Evaluation of Experience and Expectations

**DOI:** 10.7759/cureus.2461

**Published:** 2018-04-11

**Authors:** Eugene F Stautberg III, Jose Romero, Sean Bender, Marc DeHart

**Affiliations:** 1 Orthopaedic Surgery, Washington University School of Medicine, Barnes-Jewish Hospital, St. Louis, USA; 2 Orthopaedic Surgery, University of Texas Southwestern, Dallas, USA; 3 Radiology, Baylor College of Medicine, Houston, USA; 4 Orthopaedic Surgery, University of Texas Health Science Center at Houston, Houston, USA

**Keywords:** health policy, practice management, first employment opportunity, survey, orthopaedic resident education

## Abstract

Introduction

Practice management and health policy have generally not been considered integral to orthopaedic resident education. Our objective was to evaluate residents’ current experience and knowledge, formal training, and desire for further education in practice management and health policy.

Methods

We developed a 29-question survey that was divided into three sections: practice management, initial employment opportunity, and health policy. Within each section, questions were directed at a resident’s current experience and knowledge, formal training, and interest in further education. The survey was distributed at the end of the academic year through an Internet-based survey tool (www.surveymonkey.com) to orthopaedic residents representing multiple programs and all postgraduate years.

Results

The survey was distributed to 121 residents representing eight residency programs. Of those, 87 residents responded, resulting in a 72% response rate. All postgraduate years were represented. Regarding practice management, 66% had “no confidence” or “some confidence” in coding clinical encounters. When asked if practice models, finance management, and coding should be taught in residency, 95%, 93%, and 97% responded “yes,” respectively. When evaluating first employment opportunities, the three most important factors were location, operating room block time, and call. Regarding health policy, 28% were “moderately familiar” or “very familiar” with the Physician Payments Sunshine Act, and 72% were “not familiar” or “somewhat familiar” with bundled payments for arthroplasty. Finally, when asked if yearly lectures in political activities would enhance resident education, 90% responded “yes.”

Discussion and conclusion

Regarding practice management, the survey suggests that current orthopaedic residents are not familiar with basic topics, do not receive formal training, and want further education. The survey suggests that residents also receive minimal training in health policy. Residents feel that health policy will be important in their careers, and they would benefit from formal training in residency.

## Introduction

Surgical resident education has evolved from the “See One, Do One, Teach One” apprentice model to programs focusing on evidence-based medicine that includes simulation labs and a multidisciplinary team approach. Simultaneously, health policies regarding documentation, billing, and value-based payment models have increased the demands on physicians to fully manage their patients. However, orthopaedic education regarding health policy and practice management has not kept pace.

Currently, approximately 75% of new orthopaedic graduates change their first job within two years [1], suggesting that new graduates are not well prepared to critically evaluate employment opportunities. In training, residents have traditionally been sheltered from the administrative and financial interworking of the practice. After training, recent graduates are forced to learn practice management while building a patient base and treating patients autonomously for the first time. A common scenario for recently graduated residents is a transition from high-service level academic institutions to community practices that must maintain a high production level to cover growing overhead requirements in the face of declining reimbursement rates. Professional ethics and business responsibilities can result in conflicts that are resolved based on the economic forces in the community and the culture of the medical practice leadership.

A previous survey of graduating orthopedic residents showed that over 90% felt formal training in coding and billing was essential to training; however, the results were underpowered, as there was only a 25% response rate [2]. A survey of residency program chairmen and directors shows only 33% of programs included leadership training. While 96% of the programs represented included some education on at least one business topic, the depths of training were not discussed [3]. A main limiting factor of many surveys is the low response rate.

The Accreditation Council for Graduate Medical Education (ACGME) has outlined six core competencies, one of which is system-based practice. These guidelines state residents should work effectively in various health care delivery settings and coordinate patient care within the health care system (ACGME) [4]. Although these guidelines were laid out, they have not been defined further, nor has an ACGME-standardized curriculum or plan of execution been developed.

Our objective is to evaluate current orthopaedic residents’ knowledge, formal training, and desire for further training in the avenues of practice management and health policy. We hypothesize that current orthopaedic residents do not feel comfortable handling practice-management situations and are not familiar with current health policy topics. Further, we hypothesize these residents do not receive formal training and feel formal training in practice management and health policy would improve their residency education.

## Materials and methods

**Figure 1:**
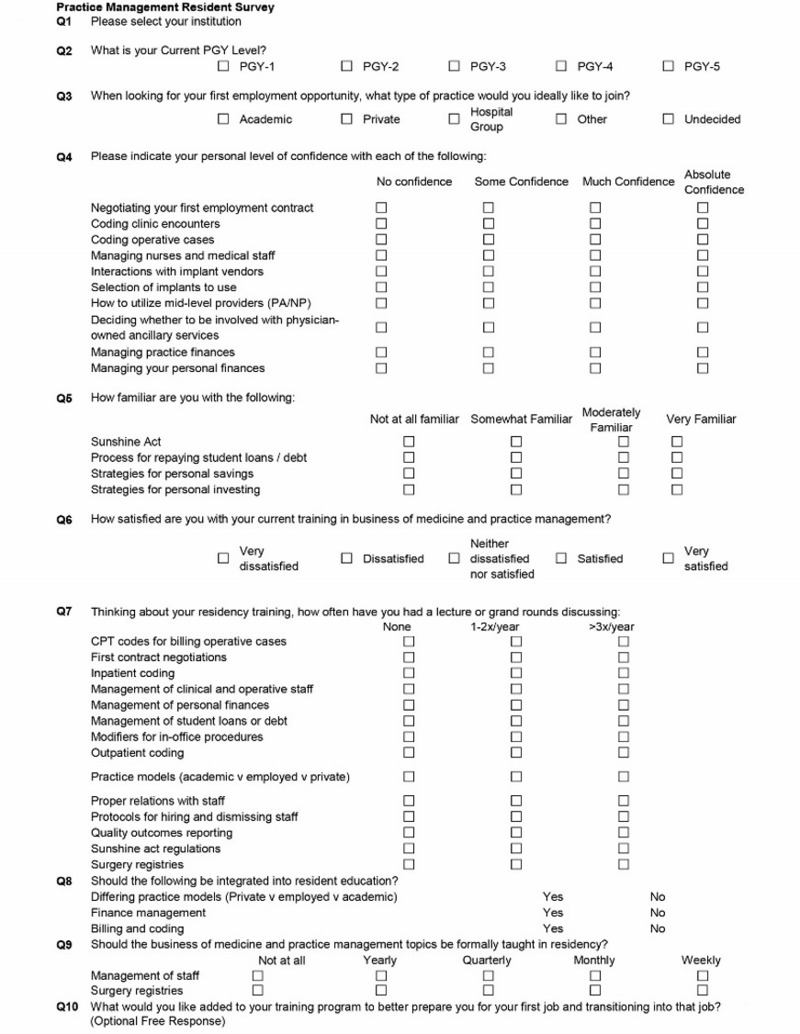
Practice management resident survey. PGY: Post-graduate year; CPT: Current Procedural Terminology® Medical Code Set (established by the American Medical Association).

We developed a 29-question survey that was divided into three sections involving practice management and health policy. The first section focused on practice management (Figure 1). Initial questions were directed at residents’ current experience and knowledge over specific topics, including coding encounters, utilizing mid-level providers, and finance management. The next set of questions evaluated a resident’s current formal training over those topics, specifically looking at the frequency of formal lectures in a resident’s education. The final series of questions gauged the resident’s interest in further education. Likert scales were used with four tags. Additionally, an optional free response question was available to clarify and expand upon answers.

**Figure 2:**
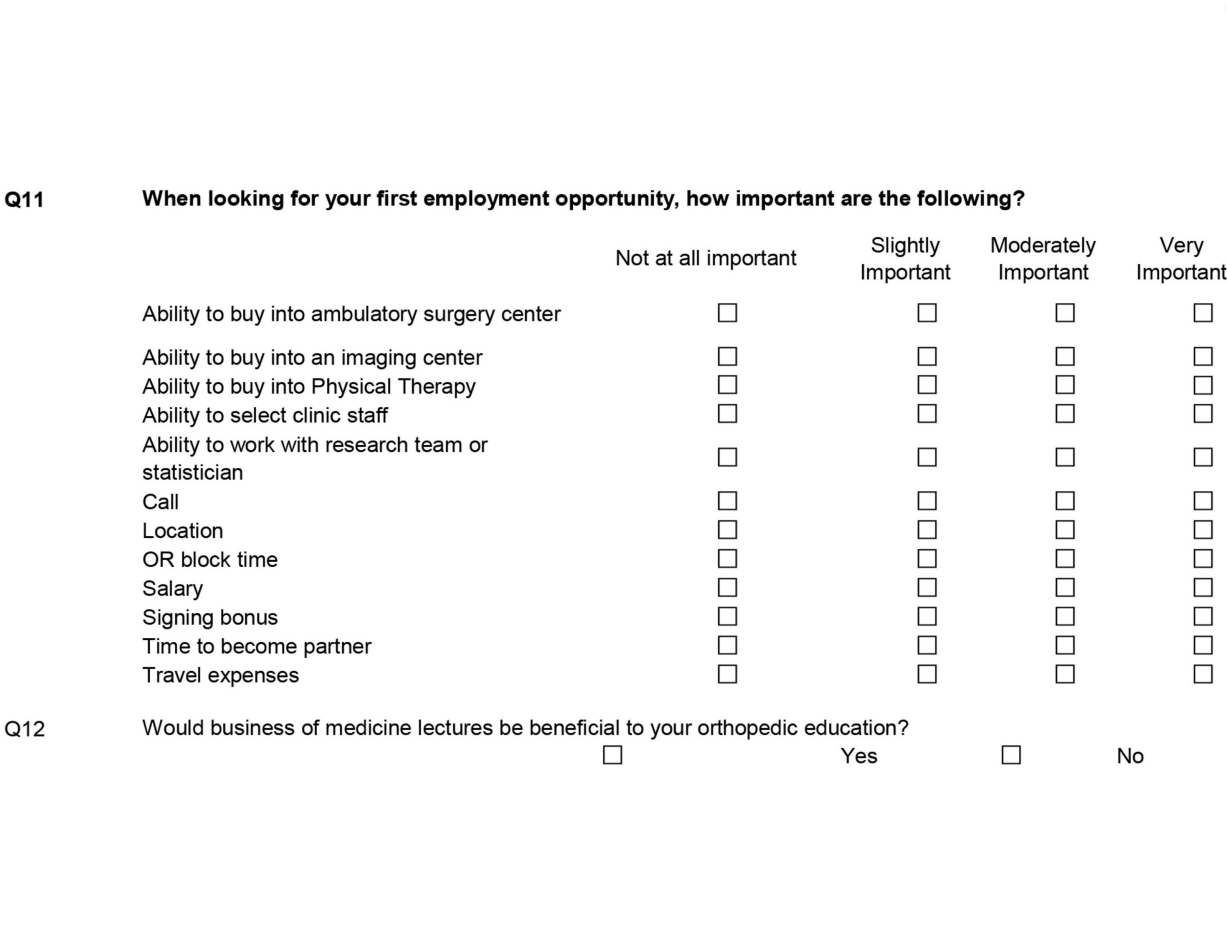
First employment opportunity survey: areas of importance. OR: Operating room.

The second section focused on first employment opportunities (Figure 2). A series of scenarios were listed and respondents were asked to state the importance of a number of factors in one’s initial job search, ranking them using four Likert scales from ‘not important’ to ‘very important’. These factors included salary, call, and operating room block time.

**Figure 3:**
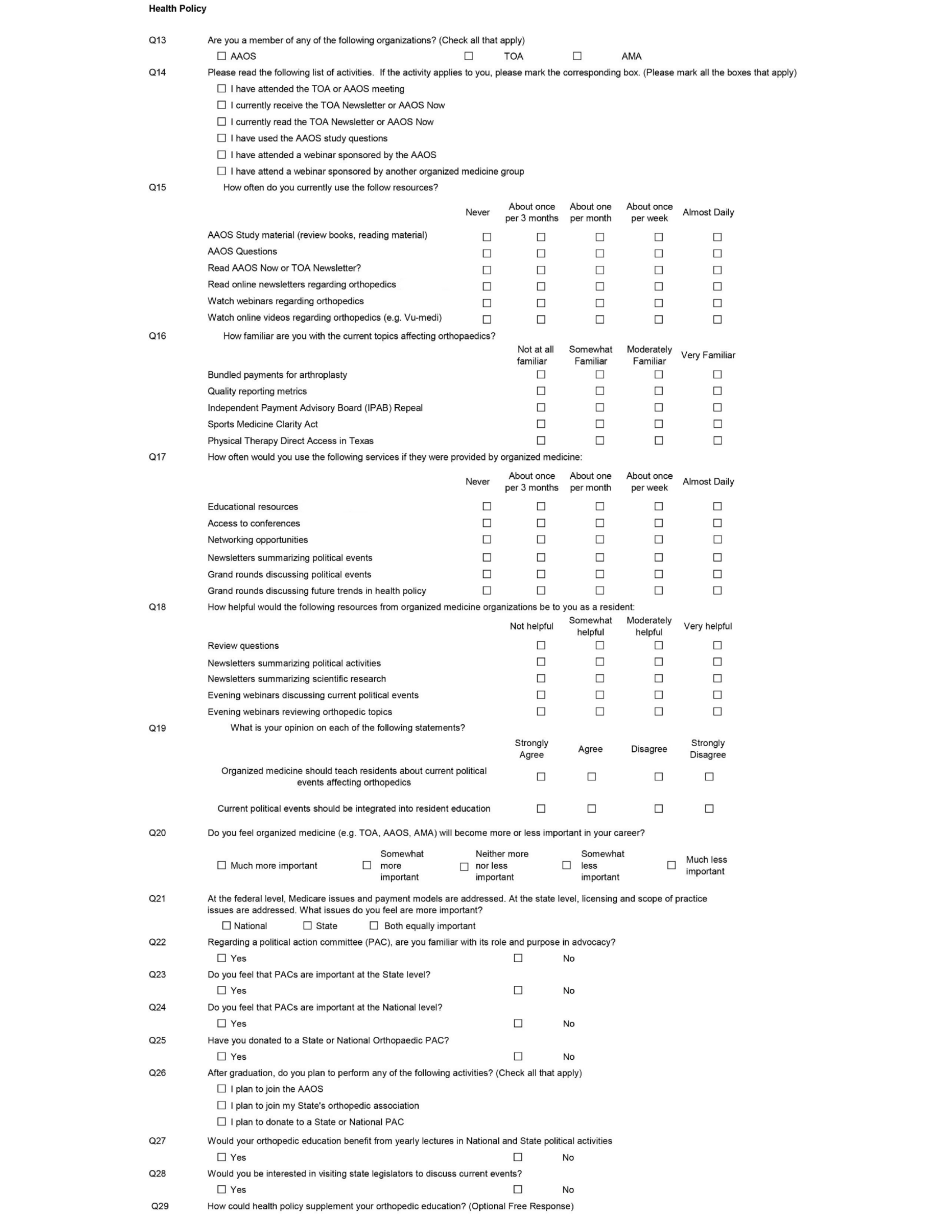
Health policy resident survey. AAOS: American Academy of Orthopaedic Surgeons; TOA: Texas Orthopaedic Association; AMA: American Medical Association.

The third section of the survey focused on health policy (Figure 3). These questions asked how involved the residents are in current organizations and how often they use content provided by these organizations. Then, using Likert scales, the survey asks how familiar the resident is with certain current legislation affecting orthopaedics. Further, residents were asked what services provided by organized medicine would supplement their education. The final questions in the health policy section asked the familiarity and involvement with the orthopaedic Political Action Committee (PAC). Another free response question allowed for expansion on any topics.

The survey was distributed during the last quarter of the academic year via a Web-based survey tool (www.surveymonkey.com) to orthopaedic residents of all postgraduate levels at four residency programs. Additionally, five residents were surveyed at an organized medicine group’s annual meeting from four additional programs. A “resident leader” was identified at each residency program to help coordinate distribution to all residents via resident email list serve and to facilitate completion of the survey. Additionally, a $15 gift card was offered to all who completed the survey. Once data was obtained, univariate statistics were calculated using frequencies.

## Results

The survey was distributed to 121 residents at eight orthopaedic residency programs. There were 87 responses, with a response rate of 71.9%. Respondents were equally distributed among post-graduate year (PGY) level after medical school, ranging from 17% (PGY-2) to 22% (PGY-4) (Table 1). Regarding the type of practice the respondents would initially like to join, 38% selected private practice, 19% selected academic, and 28% were undecided.

**Table 1:** Respondents by post-graduate year (PGY) level.

Post-graduate year	Number of respondents	Number surveyed	Percent responded	Percent of total respondents
PGY-1	18	23	78.3%	20.7%
PGY-2	15	25	60.0%	17.2%
PGY-3	17	24	70.8%	19.5%
PGY-4	19	25	76.0%	21.8%
PGY-5	18	24	75.0%	20.7%
Total	87	121	71.9%	

**Figure 4:**
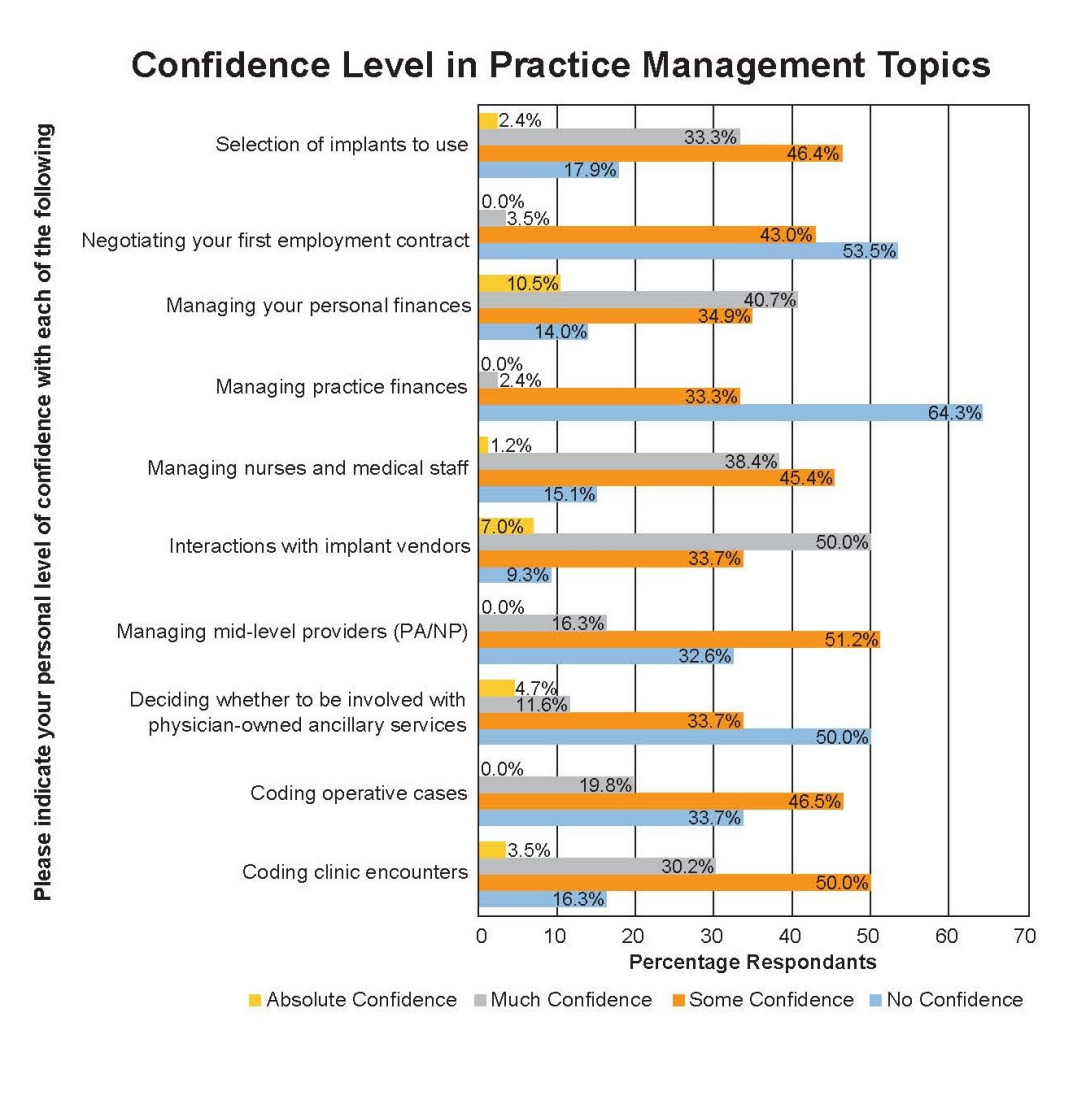
Confidence in practice management topics. PA/NP: Physician Assistant/Nurse Practitioner.

The next questions assessed resident confidence levels on varying practice management scenarios (Figure 4). Regarding coding clinical encounters, 16% had “no confidence,” 50% had “some confidence,” and 30% had “much confidence.” When asked about comfort level in negotiating a first employment contract, 53% had “no confidence,” 43% had “some confidence,” and 3% had “much confidence.”

Regarding debt and savings, results are summarized in Table 2. When asked familiarity with the Sunshine Act, 34% were “not familiar,” 38% were “somewhat familiar,” 22% were “moderately familiar.”

**Table 2:** Familiarity with practice management topics.

Question	Percent selected (%)			
How familiar are you with the following:	Not familiar	Somewhat familiar	Moderately familiar	Very familiar
Process for repaying student loans/debt	16.3%	18.6%	37.2%	27.9%
Strategies for personal investing	20.0%	34.1%	30.6%	15.3%
Strategies for personal savings	10.5%	36.1%	31.4%	22.1%
Sunshine Act	33.7%	38.4%	22.1%	5.8%

Answers concerning current lectures or grand rounds on practice management topics are summarized in Table 3. When asked about the American Medical Association’s (AMA) Current Procedural Terminology (CPT) code, 42% had 0 lectures per year, 58% had 1–2 lectures per year, and none had greater than two lectures per year. For first contract negotiations, 77% had no lectures, and 23% had 1–2 lectures per year.

**Table 3:** Evaluation of current formal training in practice management. CPT: Current Procedural Terminology® Medical Code Set (established by the American Medical Association).

Question	Percent selected (%)		
Thinking about your residency training, how often have you had a lecture or grand rounds discussing:	None	1–2x/year	>3x/year
CPT codes for billing operative cases	41.9%	58.1%	0.0%
First contract negotiations	76.7%	23.3%	0.0%
Inpatient coding	58.1%	39.5%	2.3%
Management of clinical and operative staff	88.4%	9.3%	2.3%
Management of personal finances	46.5%	53.5%	0.0%
Management of student loans or debt	66.3%	33.7%	0.0%
Modifiers for in-office procedures	69.8%	29.1%	1.2%
Outpatient coding	47.7%	50.0%	2.3%
Practice models (academic vs employed vs private)	70.6%	29.4%	0.0%
Proper relations with staff	61.6%	31.4%	7.0%
Protocols for hiring and dismissing staff	86.1%	12.8%	1.2%
Quality outcomes reporting	59.3%	33.7%	7.0%
Sunshine Act regulations	77.9%	22.1%	0.0%
Surgery registries	75.6%	22.1%	2.3%

Ninety-five percent of residents felt different practice models should be integrated into their education. Additionally, when asked if finance management and billing/coding should be integrated into resident education, 93% and 97% responded “yes,” respectively. Residents felt that techniques in the management of staff should be taught either quarterly (44%) or yearly (46%). Survey respondents felt that lectures covering surgery registries should be held either quarterly (36%) or annually (54%).

The free-response question had 16 responses covering a spectrum of topics discussed in the survey; most focused on requesting training in coding and billing, negotiating contracts, and discussions from physicians who have recently started practices. Other comments asked for training in staff management and first-contract negotiations.

Initial employment opportunity

**Figure 5:**
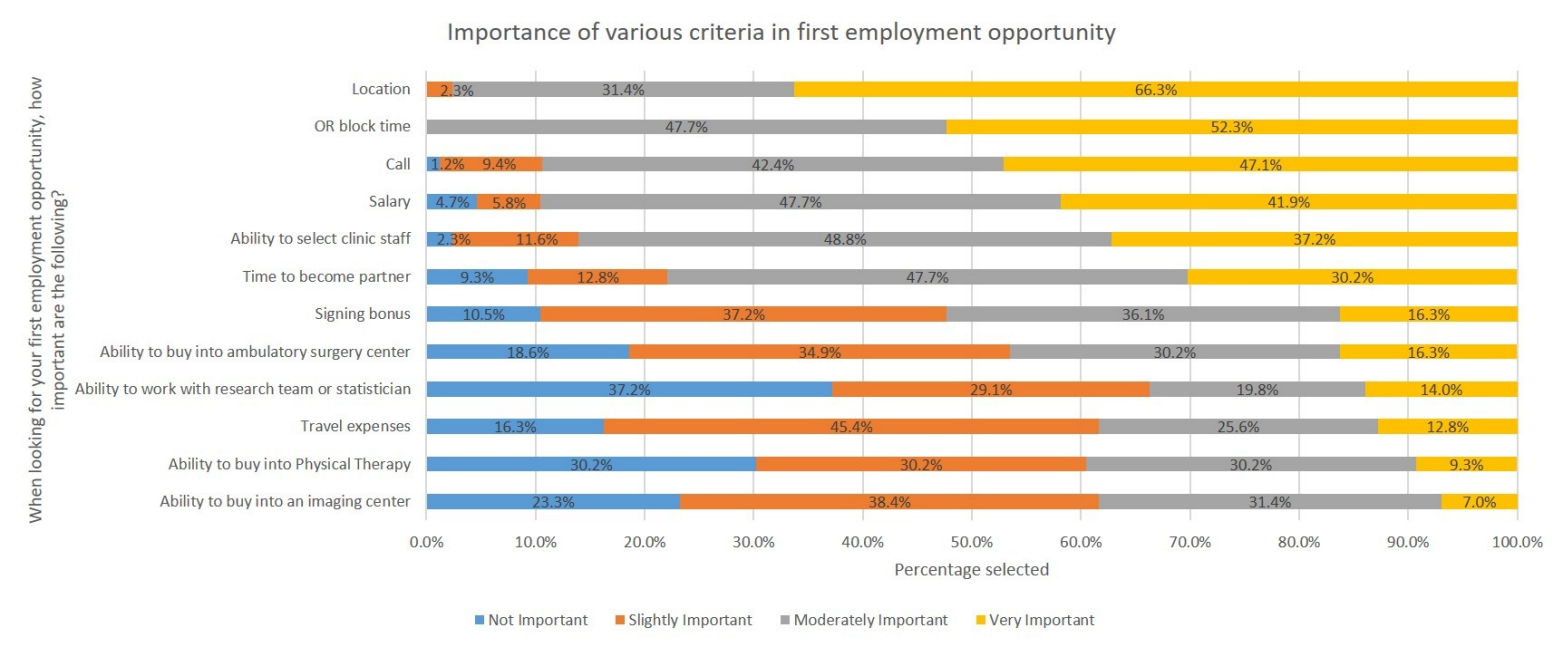
Importance of various criteria in first employment opportunity. OR: Operating room.

Residents were then asked to rank 12 variables in order of most important when looking for a first employment opportunity (Figure 5). The top five variables, from highest to lowest, were: location, operating room block time, call, salary, and ability to select clinic staff. The bottom five variables, starting from lowest, were: ability to work with research team or statistician, ability to buy into physical therapy, ability to buy into imaging center, ability to buy into a surgery center, and travel expenses.

Next, the residents were asked what material provided by organized medicine they would use most frequently (Table 4). Study questions are used weekly by 24% of residents, monthly by 28%, and quarterly by 23% of residents, respectively. Thirty-seven percent read online newsletters quarterly, and 35% read newsletters more frequently.

**Table 4:** Frequency of educational resources used. AAOS: American Academy of Orthopaedic Surgeons; TOA: Texas Orthopaedic Association.

Question	Percent selected (%)				
How often do you currently use the following resources?	Never	About once per three months	About once per month	About once per week	Almost daily
AAOS study material (review books, reading material)	9.8%	25.6%	30.5%	29.3%	4.9%
AAOS questions	22.0%	23.2%	28.1%	24.4%	2.4%
Read AAOS Now or TOA newsletter?	25.6%	34.2%	34.2%	4.9%	1.2%
Read online newsletters regarding orthopedics (AAOS or other)	28.1%	36.6%	24.4%	7.3%	3.7%
Watch webinars regarding orthopedics	41.5%	42.7%	12.2%	3.7%	0.0%
Watch online videos regarding orthopedics (e.g., Vu Medi)	18.3%	15.9%	29.3%	32.9%	3.7%

Health policy

**Figure 6:**
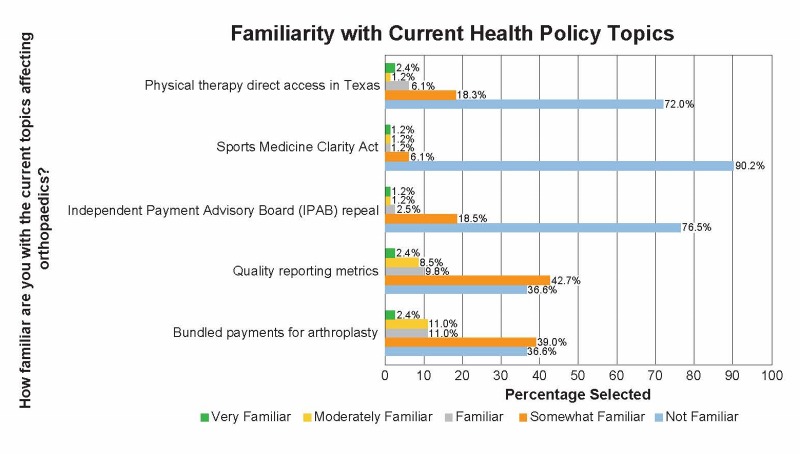
Familiarity with current health policy topics.

The survey then asked about the resident’s familiarity with various health policy topics (Figure 6). Bundled payments for arthroplasty were “not familiar” to 37%, “somewhat familiar” to 39%, “familiar” to 10%, and “very familiar” to only 2%. Regarding quality reporting metrics, 37% were “not familiar,” 43% were “somewhat familiar,” 10% were “familiar.” Seventy-two percent were “not familiar” with physical therapy direct access, and 18% were “somewhat familiar.”

The majority of residents responded that they would use conferences, networking opportunities, newsletters summarizing political events, or grand rounds on health policy once a quarter to once per month. Review questions were felt to be “very helpful” by 63% of residents. Newsletters received 6% “very helpful,” 35% “moderately helpful.” Webinars reviewing orthopaedic educational topics received 16% “very helpful” and 37% “moderately helpful.”

The residents were asked if organized medicine should teach residents about current political events affecting orthopaedics, and 25% “strongly agree,” 72% “agree,” and 2% “disagree.” Further, they were asked if current political events should be integrated into resident education, resulting in 89% selecting “strongly agree” or “agree” and 10% selecting “disagree.”

Regarding the Orthopaedic PAC, 66% were familiar and 34% were not familiar with the PAC (Table 5). Ninety-five percent feel the PAC is important at the national level, and 91% feel it is important at the state level. The majority plan to join the American Academy of Orthopaedic Surgery (AAOS) and the state organization, and 50% plan to donate to the state or national PAC.

**Table 5:** Familiarity with political action committees (PACs). AAOS: American Academy of Orthopaedic Surgeons.

Question	Percent selected (%)	
Organized medicine questions	Yes	No
Are you familiar with the role and purpose of political action committees (PACs) in advocacy?	66.2%	33.7%
Do you feel that PACs are important at the state level?	91.4%	8.6%
Do you feel that PACs are important at the national level?	95.1%	4.9%
Have you donated to a state or national orthopedic PAC?	14.5%	85.5%
After graduation, do you plan to join the AAOS?	91.5%	8.5%
After graduation, do you plan to join your state association?	85.4%	14.6%
After graduation, do you plan to donate to the state or national PAC?	50.0%	50.0%
Would your orthopedic education benefit from yearly lectures in national and state political activities?	90.2%	9.7%

Finally, 90% feel their education would benefit from yearly lectures in national and state political activities. The free-response question had six responses, the majority of which were interested in the practical application of how public policy would affect the management of their future practice and patients.

## Discussion

Physicians work at an intersection between point-of-care, hospitals, and law-makers, where they deliver care and advocate for patients [5]. As healthcare continues to evolve and hold a national spotlight, knowledge of practice management and the current political landscape may become increasingly important to physicians’ ability to deliver patient care. This survey suggests current orthopaedic residents are not comfortable with basic principles of practice management, their formal orthopaedic education does not integrate practice management topics, and residents are interested in further practice management education.

Previous surveys support these findings. Wiley et al. evaluated billing and coding knowledge of orthopaedic residents and attending surgeons [6]. They found that although 87.5% were active in coding in their practices, only 65.2% received formal training. Furthermore, 98% of residents wanted formal training in coding and billing. This survey showed over half of the residents were not comfortable with coding clinical encounters or operative cases, about half of the residents have received at least one lecture on coding, and the vast majority feel coding and billing should be integrated into resident education. With regard to practice management, the vast majority were not comfortable managing medical staff or practice finances. However, residents did have confidence interacting with implant vendors, which may be a result of the implant companies engaging residents rather than programs training residents in responsible relationships. To underscore this, a third of residents have not heard of the Sunshine Act.

With a continued shift to value-based care, physicians are in a position to make meaningful change in delivering care to their patients, but residency programs fail to provide adequate or well-defined practice management education [3]. Dyrda’s data showing that the vast number of graduates change jobs within the first two years is supported by the data in our study, where residents acknowledge that they are not comfortable with first employment contracts [1]. One can conclude that an opportunity exists to improve graduates’ early workplace experience by providing education in residency on contract negotiations and different practice models.

When considering first-employment settings, the majority selected private practice, but a large percentage was also undecided, again showing a need for education on strengths and weakness of different employment models. When evaluating factors that residents are looking for in their first job, topics that gave control to one’s daily schedule appeared to be most important, including location, operative room block time, call, and selection of clinical staff. These results show that current residents value the flow of their daily and weekly schedules to balance work and personal time. Financial factors, such as a signing bonus, salary, and travel expenses ranked in the middle to bottom of importance. This may show that initial compensation may not be as important, if they can start a practice in a location of choice with control over setting their operative schedule, call, and clinical staff. Finally, the ability to buy into ancillary services and the opportunity to work with a research team scored in the bottom third of importance. One can assume that if residents are not familiar with different practice models, they may not know the advantages and disadvantages of ancillary ownership.

The importance of politics and health policy on the day-to-day lives of physicians has increased over the last several decades. There are many avenues to get involved with health policy, including national and state specialty organizations [7]. On the national level, there has been an emphasis on increasing resident involvement in the political process. With the AMA’s Resident and Fellow Section (AMA-RFS) and the recent creation of the resident assembly at the AAOS, there has been an increase in exposure of trainees to health policy. However, this survey suggests a majority of residents are not familiar with current political events affecting orthopaedics, including bundled payments, quality reporting metrics, and physical therapy direct access. As these topics are currently being integrated into practices around the nation, there appears to be a gap in knowledge between policymakers, practicing physicians, and residents.

The current resident survey suggests methods for programs and organized medicine to best reach residents and include yearly versus quarterly lectures on health policy. There have been an increasing number of webinars created by organized medicine, but the majority of residents would not utilize webinars discussing political events. Finally, the majority do feel the current political landscape should be taught in residency.

National subspecialty organizations have worked to increase resident involvement in the political process by increasing donations to their PACs. The survey suggests that a majority of residents are familiar with the purposes of the PACs, but only a minority have donated to a PAC. Shah et al. evaluated factors influencing resident participation in PACs. From their study, the two most common barriers to donation were time constraints and inability to access PAC Web portals; however, participation in PACs increased from 10% to 95% following the introduction of faculty contribution match programs [8].

At an American Orthopaedic Association resident leadership forum, residents surveyed stated few programs provided formal practice management education, and they called for specialized curriculum in small business practice, coding/billing, and medicolegal issues [9]. There are many opportunities for future research in education and health policy [10].

Different groups have defined advocacy and business curriculums, such as the Reno Orthopaedic Center Trauma Fellowship. Their program outlines graduate-level business education over the 12-month fellowship, with multiple defined objectives [11]. One study developed an advocacy curriculum and evaluated residents before and after the curriculum was implemented [12]. Before the curriculum, only 24% of residents received any orthopaedic advocacy education. The curriculum involved a series of lectures, grand rounds, and journal clubs over advocacy topics, and after completion of the series at one institution, all residents felt learning about advocacy was important [12]. Residency programs and organized medicine groups can use these programs to outline and develop comprehensive resident-level curriculums.

The limitation of this study includes only having 87 responses from eight residency programs from one state; however, this study did have a 72% response rate. Additionally, one program had a lower response rate (36%), so the data may not represent all residents in that program. This survey included all levels of training, and one can assume a senior resident may be more familiar than an intern with the topics in question. The survey did not attempt to quantify specific practice management knowledge; rather, it asked personal interpretation of comfort levels. Further studies are needed to quantify residents’ actual knowledge in practice management and health policy.

These results paired with future research can help residency programs and organized medicine develop, implement, and evaluate practice management and health policy curriculums to fill the current void in resident education.

## Conclusions

Practice management education is only minimally taught in orthopaedic residencies, yet current residents have a strong interest in integrating practice management into their formal education. Residents are often not familiar with topics in health policy, and the large majority feels their education should include at minimum yearly discussions regarding health policy.
